# The cumulative effects and clinical safety of repeat magnetic resonance imaging on an MRI-conditional Pacemaker system at 1 .5 Tesla

**DOI:** 10.1186/1532-429X-18-S1-P204

**Published:** 2016-01-27

**Authors:** Amir K Durrani, Sarah Sandberg, Marye Gleva, Kevin W Mitchell, Matthew D Keith, Pamela K Woodard

**Affiliations:** 1 Cardiovascular Medicine, Washington University St. Louis, St. Louis, MO USA; 2 Mallinckrodt Institute of Radiology, Washington University Saint Louis, St. Louis, MO USA; 3 Cardiac Rhythm Management, Biotronik Inc., Boston, MA USA; 4 Cardiovascular Medicine, Saint Louis University, St. Louis, MO USA

## Background

We evaluated the safety and performance of an MRI conditional pacemaker after serial magnetic resonance imaging (MRI) in this multicenter, prospective, single arm study.

## Methods

The ProMRI study, a multicenter prospective trial of an MRI conditional pacemaker (PM), was approved by local institutional review boards (IRB) and all patients provided written informed consent. Patients underwent head and lumbar and cardiac or thoracic MRIs under 1.5-T conditions and five underwent additional clinical MRI exams. Discrete PM parameters, including chamber pacing capture threshold (PCT), lead impedance (LI), sensing amplitude (SA), and battery capacity (BC) were collected before phase A and immediately and 3 months post phase B. Freedom from pacing system-related serious adverse device effects (SADEs) through 1 month post-MRI served as a primary end-point. Mean changes in pacemaker function parameters, as well as threshold successes and freedom from attenuation, were analyzed for clinical relevance and statistical significance using paired t-tests, serving as additional endpoints.

## Results

In the eighty-one patients evaluated in this trial, no adverse events or SADE occurred. Statistically significant changes (p≤ 0.05) in ventricular PCT (0.034 ± 0.15 V) immediately after, and ventricular LI immediately (-18.7 ± 44.2 Ω) and 1 month post phase B (-19.8 ± 44.9Ω) were not clinically relevant. Statistically significant atrial sensing attenuation immediately (-0.27 ± 0.92 mV) and 1 month post phase B (-0.22 ± 0.92) were also clinically irrelevant.

## Conclusions

These results demonstrate the safety and performance of the ProMRI PM in patients subject to serial MRIs.

